# The bright and dark side of blue-enriched light on sleep and activity in older adults

**DOI:** 10.1007/s11357-025-01506-y

**Published:** 2025-01-17

**Authors:** Débora Barroggi Constantino, Katharina A. Lederle, Benita Middleton, Victoria L. Revell, Tracey L. Sletten, Peter Williams, Debra J. Skene, Daan R. van der Veen

**Affiliations:** 1 Chronobiology Section, Faculty of Health and Medical Sciences, University of Surrey, Guildford, UK; 2 School of Mathematics, Physics and Space, University of Surrey, Guildford, UK; 3 Present Address: Surrey Sleep Research Centre, Faculty of Health and Medical Sciences, University of Surrey, Guildford, UK; 4 Present Address: School of Psychological Sciences, Turner Institute for Brain and Mental Health, Monash University, Melbourne, VIC Australia

**Keywords:** Light exposure, Circadian rhythms, Actigraphy, Aging, Sleep, Chronobiology

## Abstract

Low indoor light in urban housing can disrupt health and wellbeing, especially in older adults who experience reduced light sensitivity and sleep/circadian disruptions with natural aging. While controlled studies suggest that enhancing indoor lighting may alleviate the negative effects of reduced light sensitivity, evidence for this to be effective in the real world is lacking. This study investigates the effects of two light conditions on actigraphic rest-activity rhythms and subjective sleep in healthy older adults (≥ 60 years) living at home. Two photon-matched lights were compared; a control white light (4000 K) and a blue-enriched white light (17000 K) at two different intensities (300–450 lx and 1100–1200 lx respectively). Participants (*n* = 36, 25 female) completed an 11-week randomized, cross-over study, comprising 1 week of baseline, 3 weeks of self-administered light exposure (2 h in the morning and 2 h in the evening), and 2 weeks of washout for each light condition. Participants completed sleep diaries, wore a wrist actigraph and a light sensor necklace, and collected urine to measure 6-sulphatoxymelatonin. Longer duration of morning blue-enriched light significantly improved rest-activity rhythm stability and decreased sleep fragmentation. More time spent above 2500 lx increased actigraphy amplitude, daytime activity, and advanced bedtime. Evening light exposure, however, increased sleep latency and lowered sleep efficiency. Our findings show morning blue-enriched light is beneficial whereas evening light should be avoided. Optimal timing of self-administered light interventions thus may offer a promising strategy to improve sleep and rest-activity rhythms in older adults in real-world settings.

**Supplementary Information:**

The online version contains supplementary material available at 10.1007/s11357-025-01506-y.

## Introduction

Aging is associated with substantial changes in sleep and circadian rhythms, including reduced sleep quality, earlier bedtimes and wake times, increased night awakenings, and lower circadian rhythm amplitude [1, 2]. These changes are partly due to age-related ocular alterations (e.g., yellowing and clouding of the lens, reduced pupil size, and fewer photoreceptors) that diminish light input to the circadian clock in the hypothalamic suprachiasmatic nuclei (SCN), providing a weaker drive for circadian entrainment [3–5]. Additionally, older adults often experience altered light exposure profiles including insufficient daytime light and excessive nighttime light, especially in care homes [6, 7]. There are also age-related impairments in mobility [8] and a decline in social contacts [9], which are important behavioral factors contributing to daytime light deprivation.

Light’s impact on physiology is influenced by its timing, intensity, duration, and wavelength, with short-wavelength blue light (420 – 480 nm) being the most potent for stimulating biological effects [10, 11]. The timing of light administration significantly impacts both the direction and magnitude of phase shifting the circadian clock, with light in the evening delaying biological rhythms whereas light in the morning advances rhythms [12, 13].

Previous studies suggest that blue-enriched light can increase daytime activity [14], reduce insomnia, and increase rest-activity amplitude [15], strengthening the rest-activity rhythm in older people [16]. These studies, however, have focused on participants with mild cognitive impairment or Alzheimer’s/dementia, living within institutions [14, 17–19], which are controlled environments and thus, can limit our understanding of the usefulness of light interventions in healthy, non-demented older adults under free-living conditions. Moreover, the few published home-based light treatment studies have typically lasted for only a few days, which may not be sufficient to yield significant impacts [20, 21]. Unfortunately, many of these studies also lack detailed descriptions of the light quality and conditions.

The current study aimed to recruit older adults with sleep problems living at home to compare the effects of two photon-matched light conditions (administered as desktop light boxes) with different spectral compositions (blue-enriched white light, 17,000 K, vs. control white light, 4000 K) at two different intensities (low intensity, 300–450 lx vs. high intensity, 1100–1200 lx) on actigraphic rest-activity rhythms, individual patterns of light exposure, and subjective sleep metrics. We hypothesized that both light conditions (blue-enriched and control white) would improve rest-activity amplitude, stability of rest-activity rhythms, and sleep quality, as well as decrease rhythm fragmentation, with stronger effects expected from the higher lux, blue-enriched light.

## Methods

### Study population

Older adults (*N* = 154) were recruited between October 2006 and November 2008 through local newspapers advertisements. Participants were 60 years of age or over, not in full-time employment (22% part-time), and with self-reported sleep problems (Pittsburgh Sleep Quality Index, PSQI > 5). Exclusion criteria included regular use of medication affecting sleep or melatonin, psychiatric/neurological history, ocular disorders, or recent travel across more than 2 time zones. A total of 36 participants were included in questionnaire analyses and 28 in actigraphy analyses (Fig. S1, Supplementary material). Informed consent was obtained and the study was approved by the University of Surrey Ethics Committee (EC/2006/57/SBMS).

### Study protocol

**Fig. 1:**
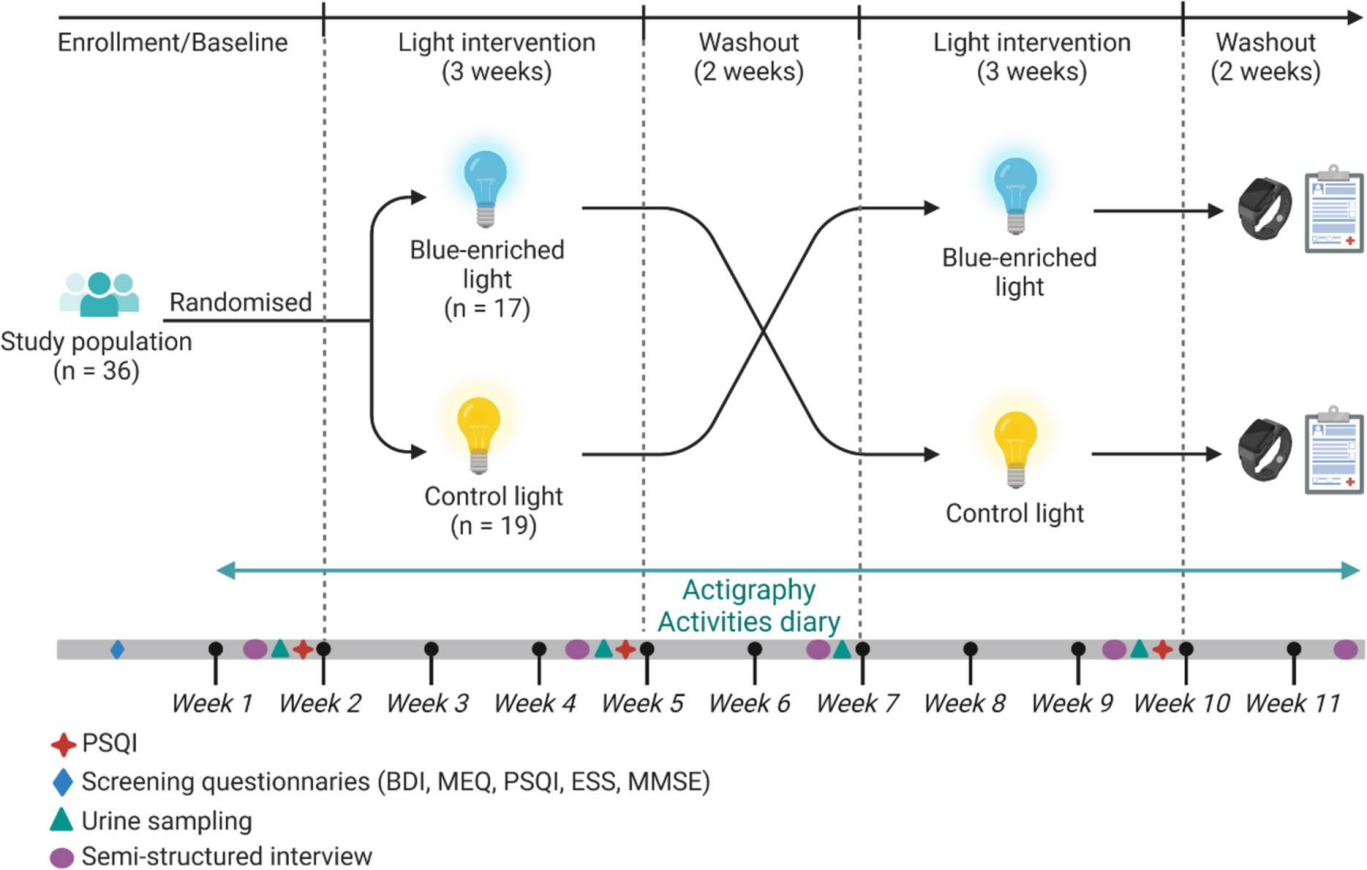
Light study protocol overview. BDI = Beck Depression Inventory; MEQ = Horne-Östberg Questionnaire; PSQI = Pittsburgh Sleep Quality Index; ESS = Epworth Sleepiness Scale; MMSE = Mini-Mental State Examination. Created with BioRender.com

The study followed a randomized, crossover design conducted over 11 consecutive weeks in the participants’ homes during the Autumn and Winter months. The study comprised one week of baseline measurements, followed by 3 weeks of exposure to either blue-enriched or control white light. After a 2-week washout, there was a second 3-week period of exposure to either blue-enriched or control white light, followed by a final two-week washout (Fig. 1).

At the end of weeks 1, 4, 6, 9, and 11, the participants’ acceptability of the light conditions was evaluated in a semi-structured interview. During weeks 1, 4, 6, and 9, participants collected sequential urine samples to measure 6-sulphatoxymelatonin (aMT6s) acrophase, the major urinary metabolite of melatonin, as a marker of circadian phase (detailed in the *Supplementary material*).

Throughout the protocol, participants wore a wrist actigraph (Actiwatch-L, AWL, Cambridge Neurotechnology Ltd., UK) on their non-dominant wrist to record rest-activity patterns and another Actiwatch-L around their neck (from get-up time to bedtime) to measure their individual light exposure.

### Light intervention

**Fig. 2:**
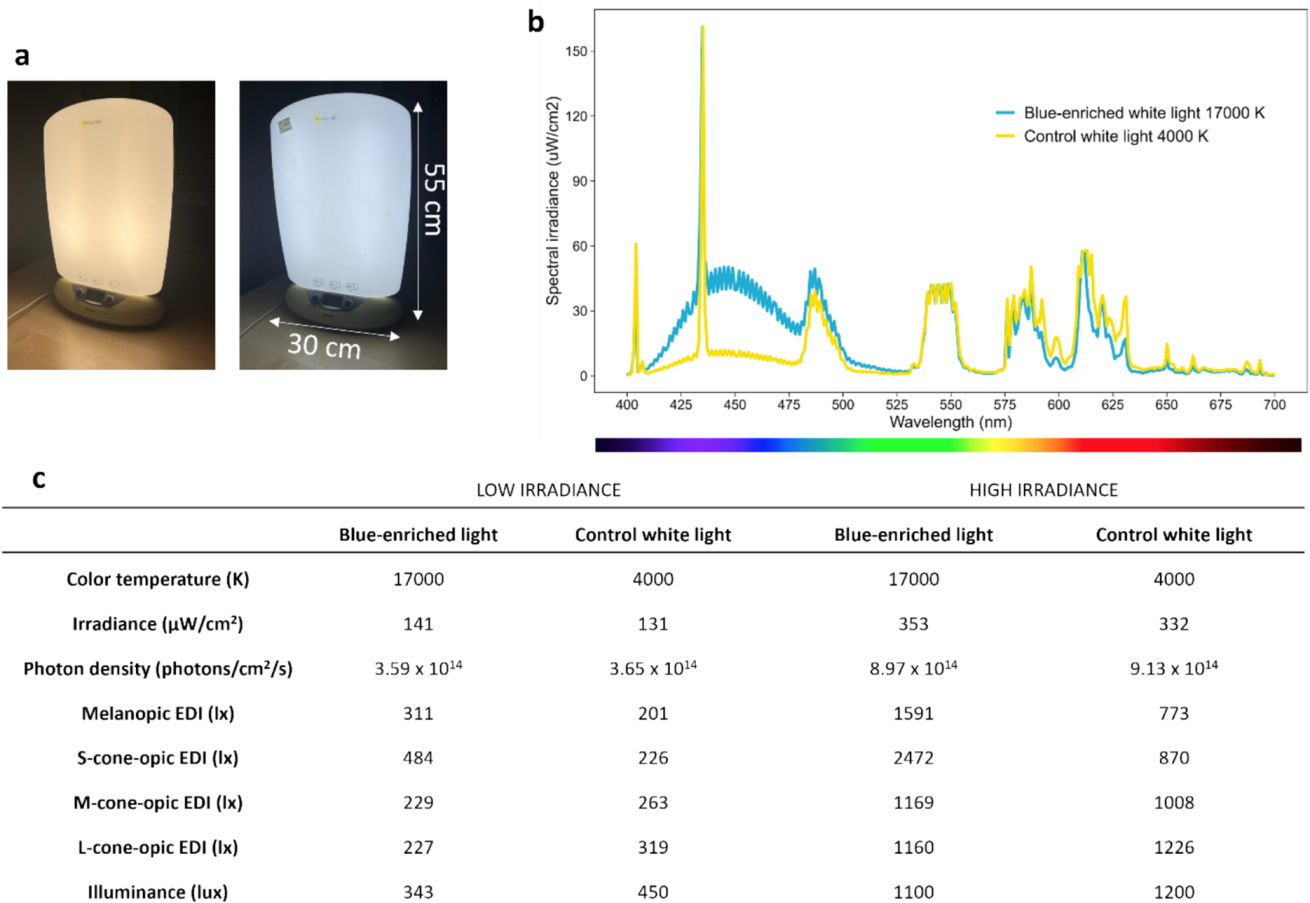
Characteristics of the administered light. **a** Light boxes used in the study (control white on the left; blue-enriched white on the right). **b** Spectral power distribution of the blue-enriched (blue line) and the control white light (yellow line). **c** Administered color temperature, intensities, photon density, melanopic EDI, and lux levels of the light administered in low and high-intensity settings. EDI (equivalent daylight illuminance) levels were calculated using the luox platform [22]

Participants self-administered light daily for 2 h each in the morning, (instructed to end before 11:00 h), and in the evening (instructed to end before 22:00 h). There were no fixed start times for the morning or evening light, however, participants were advised to start the evening light exposure after dusk. Light boxes (details on the light characteristics are shown in Fig. 2 and Supplementary material) were placed at head height and participants were asked to sit at a distance of 60 cm during the light sessions and were allowed to read, watch television, or undertake other activities. Additionally, they completed a light diary to log exposure timing/duration.

### Assessment of daily rest-activity rhythmicity and light exposure

Actigraphy data were recorded in 1-min epochs and downloaded using the Cambridge Neurotechnology software package (version 7.23). Actigraphy metrics were quantified by non-parametric circadian rhythms analysis (NPCRA) [23] using the pyActigraphy package (version 1.2.1) [24] that provided the following dependent variables: Interdaily stability (IS), Intradaily variability (IV), M10 (mean activity value for the most active consecutive 10 h of a daily profile), L5 (mean activity count of the 5 least active hours of a daily profile), Relative amplitude (RA), and onset of M10. Sleep fragmentation (kRA) was also calculated via probabilistic state transition model [25]. Spans of missing data (i.e., periods when the actigraphs were not worn) were excluded by applying a mask (i.e., spurious inactivity periods were not included in the analyses).

Light data from the necklace sensor were converted to a logarithmic scale (log10 + 1) and NPCRA was also conducted to calculate light exposure metrics using the pyLight module [26]. Additionally, we computed total time spent in light levels above 250 lx as this is the minimum level of daylight exposure advised [27] and the total time spent above 2500 lx as an estimate of outdoor light exposure over the 11-week protocol. The light metrics were incorporated as independent variables.

### Self-reported sleep variables

Participants completed daily sleep diaries to record their bedtime, sleep latency, wake-up time, sleep efficiency (percentage obtained by dividing sleep duration by total attempted sleep duration), and naps. Participants also completed the PSQI as an assessment of subjective sleep quality at baseline, at the end of the light intervention sessions at week 4 and week 9, and two weeks after the end of the study (“post-study” week 13).

### Statistical Analyses

Data are reported as means and standard deviations (SD), or median and interquartile range (IQR) as appropriate. Weekly mean values were obtained for each outcome for each participant throughout the protocol. For analysis, a total mean value was calculated for the three weeks of blue-enriched light/control light interventions and the two weeks of washout.

For each outcome, a mixed linear model with participants as repeated measures was carried out. Models included fixed effects for light conditions (blue-enriched vs. control), intensity level (low vs. high), and sequence (blue light first), with baseline measures (week 1) as covariates to control for individual variability at the start of each condition. To check for cross-over study related effects, a sequence categorical variable (blue first) was included. For each of these models, an initial exploratory analysis was conducted to identify significant independent variables. Based on the results, final models were constructed retaining only the fixed variables and significant predictors to minimize potential biases introduced by missing data and to reduce type I errors. Missing data were handled by fitting models using restricted maximum likelihood estimation (REML), which is a robust method for dealing with missing observations under the assumption of data missing at random [28]. To maintain appropriate levels of degrees of freedom, the final covariates were selected based on an initial exploratory analysis to identify relevant covariates. Based on these exploratory results, final models were constructed retaining the fixed variables and significant covariates to reduce type I errors.

These analyses were done for all parameters except the PSQI data, as no washout PSQI values were calculated due to the 4-week interval required for PSQI testing, preventing baseline adjustments. A paired t-test compared baseline PSQI with the PSQI score after the study’s completion (week 13). Additionally, we conducted a linear regression analysis using chronotype as a continuous outcome measure based on the MEQ scores to investigate the relationship between chronotype and light exposure timing. Mixed linear models were performed using IBM SPSS Statistics (Version 29.0.1.0) and all other analyses were completed in RStudio (Version 2023.03.1 + 446).

Power analysis for repeated measures ANOVA was conducted using G*Power 3.1 to determine the sample size needed to detect a medium effect size (*f* = 0.25 with 80% power) at an alpha level of 0.05. For a two-group comparison (i.e., Blue-enriched light vs. Control light), the analysis indicated a required total sample size of 24 participants. With 28 participants included for actigraphy analysis, we achieved a power of 87%, providing adequate statistical power.

## Results

### Characteristics of the study population

In total, 36 individuals were included in the study (Table 1), with a median age of 66.5 years; 25 (69%) individuals were female. Participants exhibited evidence of self-reported sleep problems (PSQI > 5), with a median PSQI score of 12.0. Participants in the control and blue light first groups had similar characteristics, except for a 3-year higher median age of 68 in the blue-enriched light first group (Mann–Whitney U test: *p* = 0.04).

**Table 1:** Participant characteristics at baseline

	Overall^1^ *N* = 36	Control light first^1^ *N* = 19	Blue-enriched light first^1^ *N* = 17
Age years	66.5 (62.8, 69.3)	65.0 (61.5, 68.0)*	68.0 (65.0, 72.0)*
Sex			
Female	25 (69%)	14 (74%)	11 (65%)
Male	11 (31%)	5 (26%)	6 (35%)
BDI	8.5 (6.0, 14.0)	10.0 (7.0, 16.5)	8.0 (6.0, 13.0)
ESS	6.0 (3.8, 9.0)	8.0 (5.0, 9.0)	5.0 (3.0, 8.0)
MEQ	61 (54, 66)	61 (52, 66)	61 (55, 66)
PSQI	12.0 (10.7, 15.0)	11.0 (10.5, 14.5)	12.0 (11.0, 15.0)
MMSE	29.5 (28.2, 30.0)	29.0 (29.0, 30.0)	30.0 (28.0, 30.0)
Intensity level			
Low intensity	*N* = 15 (42%)	*N* = 8 (42%)	*N* = 7 (41%)
High intensity	*N* = 21 (58%)	*N* = 11 (58%)	*N* = 10 (59%)

^1^Median (IQR), n (%); *BDI* beck depression inventory, *ESS* epworth sleepiness scale, *MEQ* horne-östberg questionnaire, *PSQI* pittsburgh sleep quality index, *MMSE* mini-mental state examination; * *p* < 0.05 (t-test); Low-intensity level: 300–450 lx; High-intensity level: 1100–1200 lx

### Light intervention exposure times

**Fig. 3:**
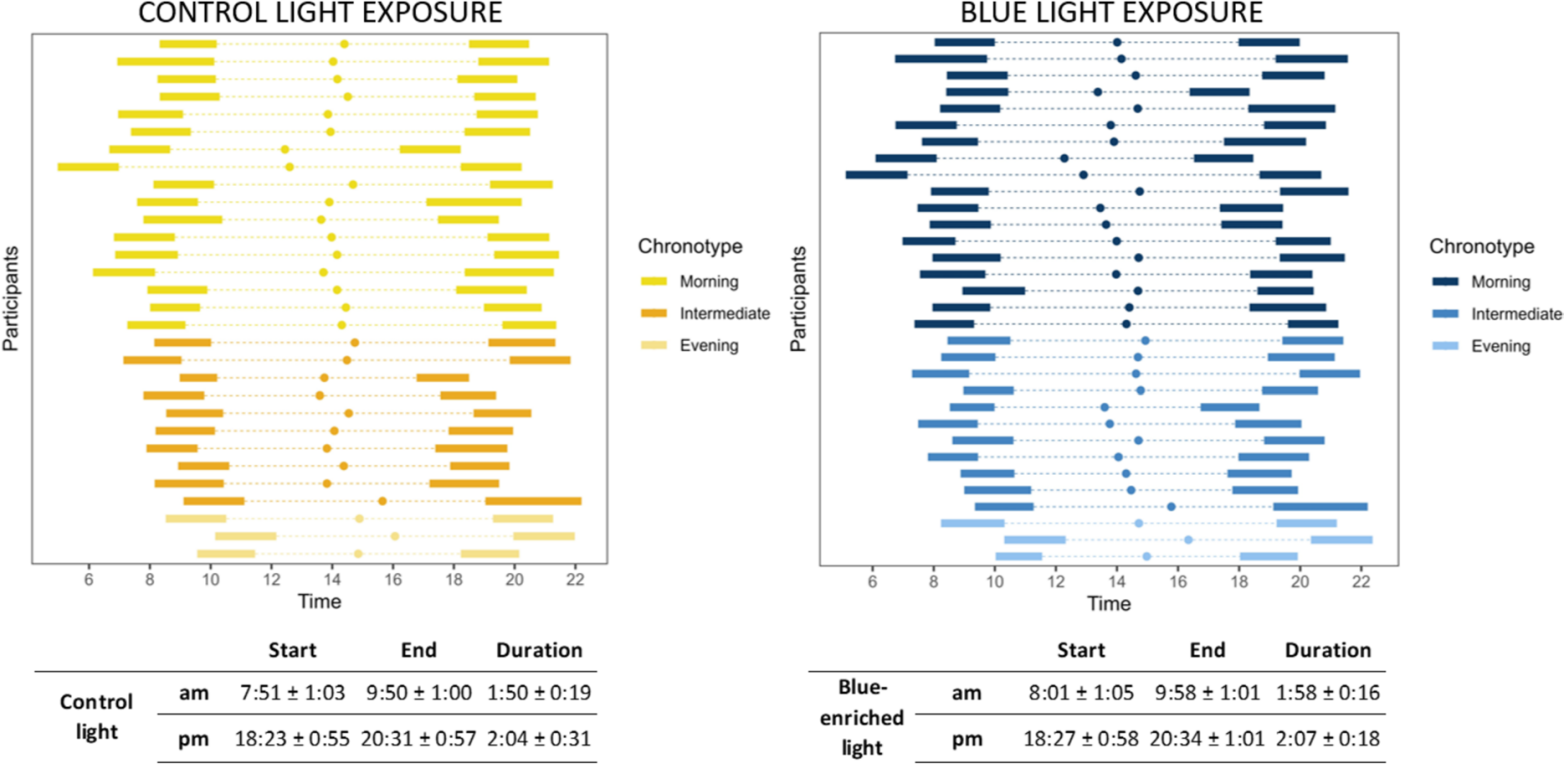
Self-reported light exposure times sorted by chronotype. Each plot displays segments representing mean light exposure times for the individual participants under the two light conditions: the control light exposure (depicted in yellow) and the blue-enriched light exposure (depicted in blue) grouped by MEQ diurnal preference (chronotype). The dots within each plot indicate each participant's midpoint of light exposure (calculated from the start of morning exposure to the end of evening exposure). Below each figure, the mean (± SD) timing and duration (hours) of the light exposure sessions in the morning (am) and evening (pm) are presented

The self-reported start and end times for the self-administered light exposure varied between each participant and are shown in Fig. 3. Paired t-tests did not reveal any significant differences in the timing or duration of light exposure between the two light conditions in either the morning or evening. During the three weeks of light exposure for each condition, the mean (± SD) duration of morning control light exposure was 1.9 h ± 0.31 (with total mean exposure of 37.01 h ± 5.7), evening control light exposure was 2 h ± 0.52 (total mean exposure of 39 h ± 3.9); mean duration of morning blue light exposure was 1.96 h ± 0.26 (total mean exposure of 35.0 ± 6.4) and mean evening blue light exposure was 2.1 h ± 0.3 (total mean exposure og 37.82 h ± 7.29).

**Fig. 4:**
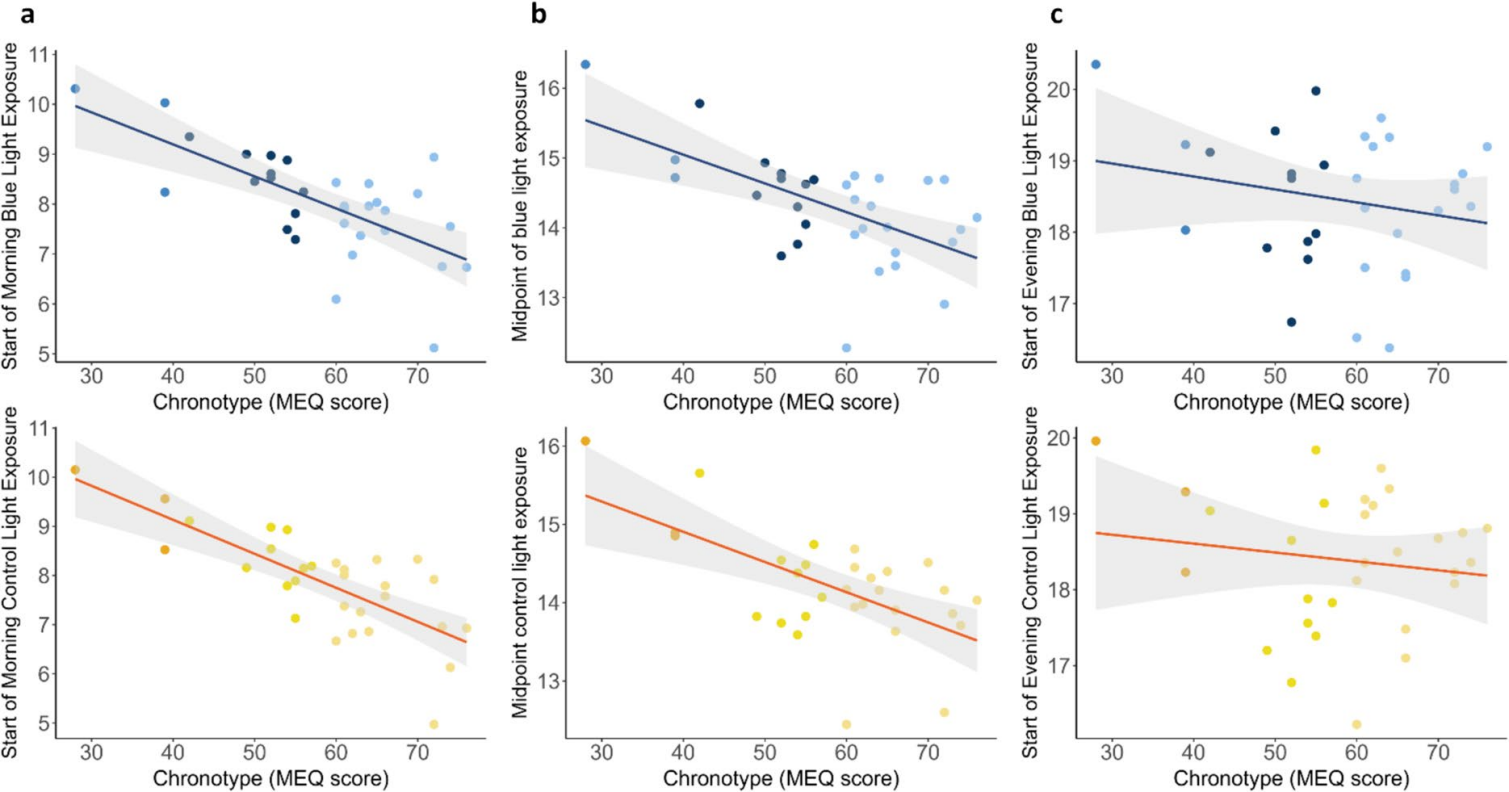
Relationship between chronotype (MEQ score) and the timing of light exposure. **a** Scatter plots showing the relationship between chronotype and the start of morning blue-enriched and control light exposure. **b** Scatter plots illustrating the relationship between chronotype and midpoint of light exposure for both blue-enriched and control light. Grey areas represent the confidence interval for the associations. **c** Scatter plots showing the relationship between chronotype and the start of evening blue-enriched and control light exposure

To investigate the relationship between chronotype and the timing of light exposure, we conducted a linear regression analysis. Chronotype significantly contributed to predicting the start of both the morning blue-enriched light (F = 25.78; R^2^ = 0.44; *p* = 1.87^e−05^) and the morning control light (F = 35.45; R^2^ = 0.54; *p* = 2.07^e−06^), with individuals with a morning chronotype starting their light exposure earlier (Fig. 4a). The same relationship extended to the midpoint of light exposure for both blue-enriched (F = 16.43; R^2^ = 0.33; *p* < 0.01) and control light (F = 16.44; R^2^ = 0.34; *p* < 0.01) (Fig. 4b). However, chronotype did not influence the start of the evening blue-enriched (F = 1.37; R^2^ = 0.01; *p* = 0.25) or control light (F = 0.59; R^2^ =—0.01; *p* = 0.44) (Fig. 4c).

Participants’ perceptions of the lighting conditions were gathered through semi-structured interviews. As summarized in Table S1 (Supplementary material), five participants made positive comments about the blue-enriched light, while four participants expressed negative opinions. For the control light, six participants made a positive remark, and two participants shared negative feedback.

### Effects of the light conditions on objective measures of daily rest-activity rhythms

We first tested whether the light intervention would improve objective measures of sleep and rest-activity compared to baseline. Overall, the repeated measures mixed linear models revealed that most actigraph parameters significantly changed from baseline to the light exposure conditions. During baseline, the interdaily stability (IS), intradaily variability (IV), relative amplitude (RA), M10, and sleep fragmentation (kRA) were significantly higher compared to the light intervention periods (IS: F = 57.52; *p* < 0.01; IV: F = 215.99; *p* < 0.01; RA: F = 105.75; *p* < 0.01; M10: F = 144.96; *p* < 0.01; kRA: F = 9.99; *p* < 0.01).

Next, we focused on our primary objective of testing the hypothesis that sleep and actigraphic parameters would show the greatest improvement with the blue-enriched light of high intensity compared to the control white light of lower intensity. Most actigraph parameters were significantly improved by the blue-enriched light exposure compared to control light exposure (Fig. S2).

**Fig. 5:**
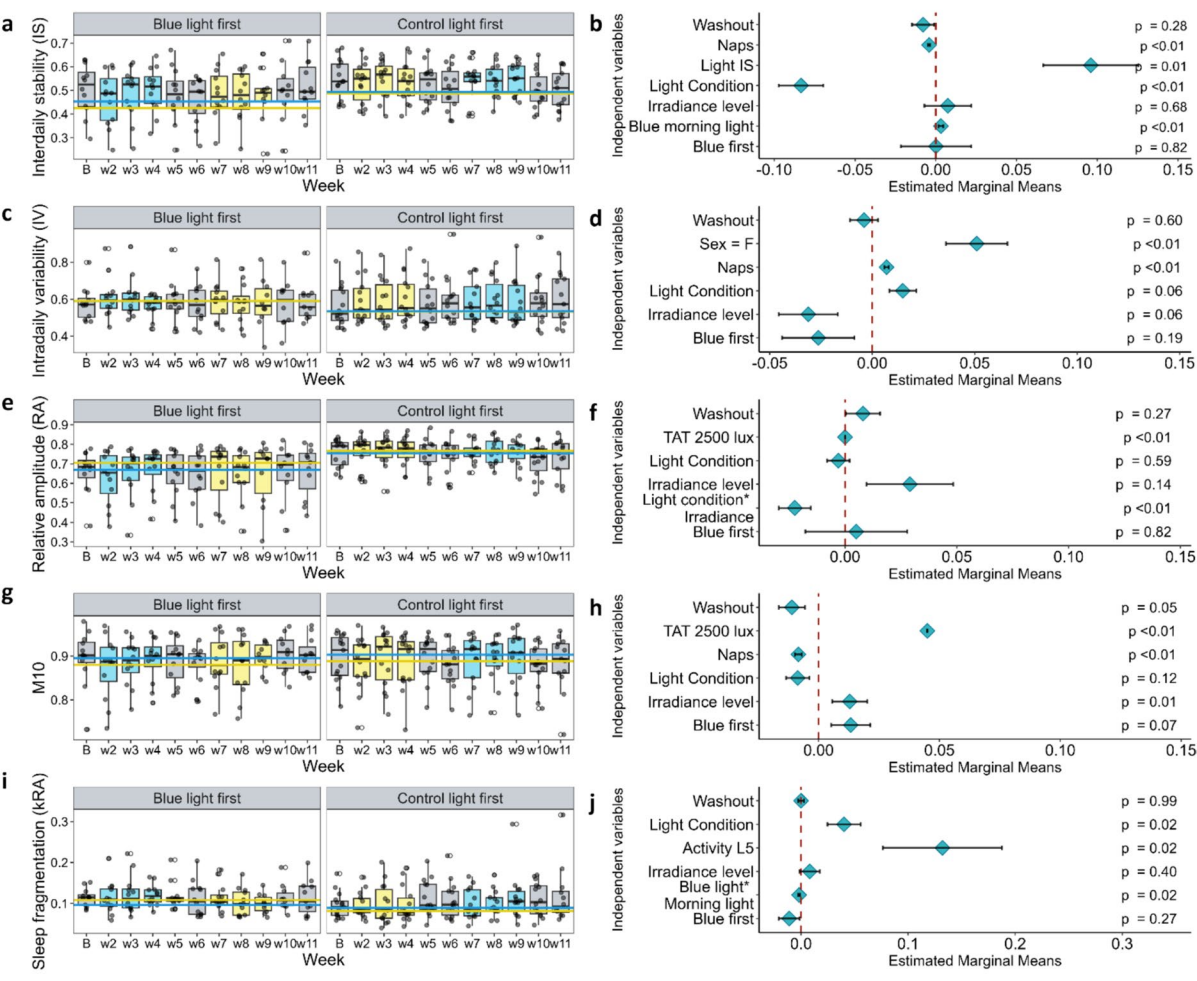
Mixed linear models results for the following outcomes: interdaily stability (IS) **a**, **b**, intradaily variability (IV) (**c**, **d**), relative amplitude (RA) (**e**, **f**), M10 (**g**, **h**), sleep fragmentation (kRA) (**i**, **j**). Boxplots: panels a, c, e, g, and i display weekly measures of each outcome for the blue-enriched light first group (first subplot in the left panel) and control light first group (second subplot). The boxplots show individual participants as grey dots, with open dots indicating outliers. Solid lines represent the median of each outcome for blue-enriched light exposure (blue line) and control white light exposure (yellow line). Forest plots in the right panel: panels b, d, f, h, and j illustrate each independent variable's estimated effect sizes, standard errors, and corresponding p-values associated with each outcome

First looking at the regularity of activity timing, we observed that participants exhibited reduced interdaily stability (activity IS) during blue-enriched light exposure compared to control light exposure (F = 26.24, *p* = 0.002). However, both a longer total duration of morning blue light exposure and increased stability of this light exposure (Light IS) significantly increased the activity IS (F = 16.09, *p* = 0.004; F = 7.36, *p* = 0.008) (Fig. 5a and b). With an increasing number of reported naps, the activity IS was reduced (F = 30.87, *p* < 0.01), and activity IV was increased (F = 7.36; *p* = 0.009) (Fig. 5c and d). Irrespective of light conditions, we observed that females exhibited a higher intradaily variability (activity IV) (F = 9.9; *p* = 0.006).

The mixed linear models also revealed a significant interaction between light condition and light intensity on activity relative amplitude (RA), with blue-enriched light exposure and low-intensity conditions both associated with lower RA (F = 11.18; *p* < 0.01). Moreover, higher RA was associated with more time spent above 2500 lx (TAT 2500 lx) across the day (Fig. 5e and f). A non-significant trend towards a higher relative amplitude was also observed with time spent above 250 lx (F = 3.45; *p* = 0.07) (Table S2, Supplementary material).

We did not observe significant effects of the light conditions and intensities on total activity in M10. However, we did see higher M10 activity levels when participants spent more time above 2500 lx (F = 25.42; *p* < 0.01). In addition, lower M10 activity levels associated with a higher number of naps (F = 40.99; *p* < 0.01) (Fig. 5g and h). We observed a non-significant trend towards higher M10 activity levels with more time spent above 250 lx (Table S3, Supplementary material).

The data also expose a significant effect of light conditions on sleep fragmentation (kRA), with blue-enriched light exposure associated with a higher kRA (F = 6.73; *p* = 0.01). Similarly, for activity stability, the mixed linear models revealed a significant interaction between light condition and light exposure time, in which a longer duration of blue light exposure in the morning was associated with lower sleep fragmentation (F = 5.05; *p* = 0.01). Higher kRA was also significantly associated with more activity at night (measured through L5 activity) (F = 5.65; *p* = 0.02) (Fig. 5i and j).

For the timing of M10 onset, there were no significant effects of light condition or intensity. Lastly, there were no significant effects of light conditions or light irradiance on 6-sulphatoxymelatonin (aMT6s) acrophase. The mixed linear models did not reveal significant effects related to the sequence of the light intervention for the outcomes mentioned above, meaning that there were no carry-over effects.

### Effects of the light conditions on subjective sleep variables

We tested whether subjective assessments of sleep would be affected by the light conditions, light irradiance, time spent above 250 and 2500 lx across the day, and duration of light exposure in the evening (Fig. S3 and Table S4). Shorter sleep latency was significantly associated with lower light intensity (F = 7.92; *p* = 0.008), while longer sleep latency was associated with higher duration of light exposure in the evening (F = 4.07; *p* < 0.001). Earlier bedtimes were significantly associated with more time above 250 lx (F = 11.07; *p* = 0.002) and more light above 2500 lx (F = 9.50; *p* = 0.004). Lastly, lower sleep efficiency was significantly associated with longer evening exposure duration (F = 22.55; *p* = 0.003), and higher sleep efficiency was significantly associated with more time spent above 2500 lx during the day (F = 6.18; *p* = 0.01). Sleep duration was not affected by the light conditions or intensities. Importantly, the sequence of light intervention did not affect subjective sleep measures.

We also tested whether the timing of light exposure would influence subjective sleep measures. Later start of morning exposure to both light conditions was significantly associated with later bedtime (F = 4.69; *p* = 0.01) and lower sleep efficiency (F = 6.49, *p* = 0.04).

When comparing baseline PSQI with PSQI on week 13, sleep quality significantly improved between baseline and after completing the entire protocol (t = 3.20; *p* = 0.003) (Fig. S4). We investigated whether any specific component of this questionnaire would account for this difference. The sleep quality component (“During the past month, how would you rate your sleep quality overall?”) significantly improved when comparing PSQI baseline with PSQI week 13 (t = 2.90; *p* = 0.008) (Fig. S5).

## Discussion

This study is the first to examine self-administered light supplementation’s effects on rest-activity timing in older, non-demented adults in real-world settings. We used photon-matched light conditions for 2 h in the morning and evening, linking both light characteristics and timing to rest-activity and sleep. Our findings reveal that longer duration of morning blue light increases rest-activity stability (IS) and decreases sleep fragmentation, while longer evening light increases sleep latency and decreases sleep efficiency. Moreover, time spent outdoors (measured as time spent above 2500 lx) enhances amplitude (RA) and daytime activity (M10), as well as eliciting an earlier bedtime. These findings provide strong evidence that exposure to blue light in the morning and increased outdoor light exposure significantly improves daily rest-activity rhythmicity and sleep quality. Also, the effectiveness of light supplementation seems to be strongly related to participant compliance, supporting self-administered light exposure as an effective real-world intervention for alleviating sleep problems in this population.

Few studies have examined light interventions on actigraphic measures of rest-activity stability (i.e., interdaily stability, IS) and fragmentation (i.e., intradaily variability, IV; sleep fragmentation, kRA) in healthy older adults. Most previous studies have focused on older adults with mild to severe cognitive impairments in assisted-living institutions [14, 17, 29]. These studies did not observe significant rest-activity stability improvements following bright light intervention, although significant improvements were found in other rest-activity parameters [17] and subjective sleep [14, 29]. Others found decreased IV after bright light therapy [18, 30] or reduced IV only early in the intervention, with the effect disappearing in later weeks [15, 31].

A key finding of this study is that a longer duration of morning blue light significantly increased interdaily stability (IS) and decreased sleep fragmentation (kRA), but these positive effects were negated if the same light was used in the evening. This aligns with previous findings that morning blue-enriched light enhances the robustness of the entrainment between internal rhythms with environmental cues, as indicated by higher IS, reflecting greater consistency of rest-activity rhythms over multiple days [18, 32–35].

The positive effects of morning light exposure may also be driven by changes in homeostatic sleep processes, as it has been reported that not only the duration of prior wakefulness but also the experienced illuminance during daytime affects the homeostatic drive for sleep [36]. Morning blue-enriched light may have increased the signal for wakefulness during the day, increasing sleep pressure and the homeostatic drive for sleep in the evening, thus improving sleep consolidation.

When considering the morning and evening blue light conditions as a whole, this was associated with decreased interdaily stability (IS), higher sleep fragmentation (kRA), and lower relative amplitude (RA). These associations can be explained by the observed relatively late-night exposure to blue-enriched light, occurring around 22:00 h. Kim et al. [37] have reported that light at night (measured with actigraphic L5) associated with lower interdaily stability (IS) in older adults. Hopkins et al. [14] also found a significant relationship between blue-enriched light and higher activity during sleep (L5). These effects could be attributed to the acute alerting effect produced by blue-enriched light that adversely affected their sleep [38]. Moreover, nighttime light exposure has already been shown to suppress circadian amplitude [39, 40]. Supporting this theory, we found that more daytime light exposure (above 2500 lx) increased rest-activity amplitude, strengthening circadian entrainment.

The light supplementation did not affect levels of daytime activity (M10), however, more time spent above 2500 lx was significantly associated with higher M10 activity levels. This activating effect of light has also been demonstrated in previous studies [14, 17, 41].

We found no differences in the M10 onset time and 6-sulphatoxymelatonin (aMT6s) acrophase time between the light conditions. Previous studies suggested that age-related reduction in light sensitivity affects neuroendocrine responses [42–44]. One study investigating the phase-shifting responses to light showed that older adults were significantly advanced in some phase markers (cortisol, sleep, aMT6s onset) but not in aMT6s acrophase after treatment with 3000 lx white light [45]. Other studies demonstrated the phase-shifting response to light is not significantly affected by age [42, 46, 47]. Thus, findings on the attenuation of light responses with aging are mixed.

The amount of time spent above 250 lx and 2500 lx was significantly associated with earlier bedtimes. This finding is in agreement with Didikoglu et al. [48] who demonstrated earlier sleep timings on days with more natural light and more robust daily patterns of light exposure in a cohort of UK adults. Additionally, we found that longer evening light exposure was significantly associated with lower sleep efficiency and higher sleep latency. While evening light may benefit older adults with early evening sleepiness [49], such treatment may not improve sleep quality in this population.

This study has shown that light supplementation for healthy, older adults in their homes has measurable positive effects on several parameters of sleep and activity. Importantly, our findings show that the positive effects are associated with a longer duration of light exposure in the morning and are negated if the same light is used in the evening, as may occur when light exposure is self-administered. It provides important evidence on the feasibility of light interventions to improve sleep and rest-activity rhythms in older adults and highlights the feasibility and benefits of morning light supplementation and daytime light exposure in a real-world setting. The current sample size provides a power over 80% giving confidence in our findings. Nonetheless, future studies should aim to replicate these findings with larger sample sizes and in different settings.

## Supplementary Information

Below is the link to the electronic supplementary material.

Supplementary file1 (DOCX 526 KB)

## Data Availability

The data sets generated and analyzed in this study are available from the corresponding authors upon reasonable request and where informed consent allows.
