# Supplementary material for: The bright and dark side of blue-enriched light on sleep and activity in older adults
Source: GeroScience. 2025 Jan 17;47(3):3927–39. doi: 10.1007/s11357-025-01506-y (PMC12181448; doi:10.1007/s11357-025-01506-y)
Supplement: Supplementary file 1 — Supplementary file1 (DOCX 526 KB) [file 11357_2025_1506_MOESM1_ESM.docx]

**The bright and dark side of blue-enriched light on sleep and activity in older adults**

*D**ébora Barroggi Constantino^1*^ MSc, Katharina A. Lederle^1*^ PhD, Benita Middleton^1^ PhD, Victoria L. Revell^1a^ PhD, Tracey L. Sletten^1b^ PhD, Peter Williams^2^ MSc, Debra J. Skene^1^ PhD, Daan R van der Veen^1^ PhD*

**^1^**Chronobiology Section, Faculty of Health and Medical Sciences, University of Surrey, Guildford, UK

**^2^**School of Mathematics, Physics and Space, University of Surrey, Guildford, UK

* These two authors contributed equally to this work.

**^a^**Current address: Surrey Sleep Research Centre, Faculty of Health and Medical Sciences, University of Surrey, Guildford

**^b^**Current address: School of Psychological Sciences, Turner Institute for Brain and Mental Health, Monash University, Melbourne, Victoria, Australia

**Corresponding authors:**

Dr. Daan R van der Veen

University of Surrey, Guildford, Surrey, United Kingdom

[d.vanderveen@surrey.ac.uk](mailto:d.vanderveen@surrey.ac.uk)

Débora Barroggi Constantino

University of Surrey, Guildford, Surrey, United Kingdom

[d.constantino@surrey.ac.uk](mailto:d.constantino@surrey.ac.uk)

**SUPPLEMENTARY MATERIAL**


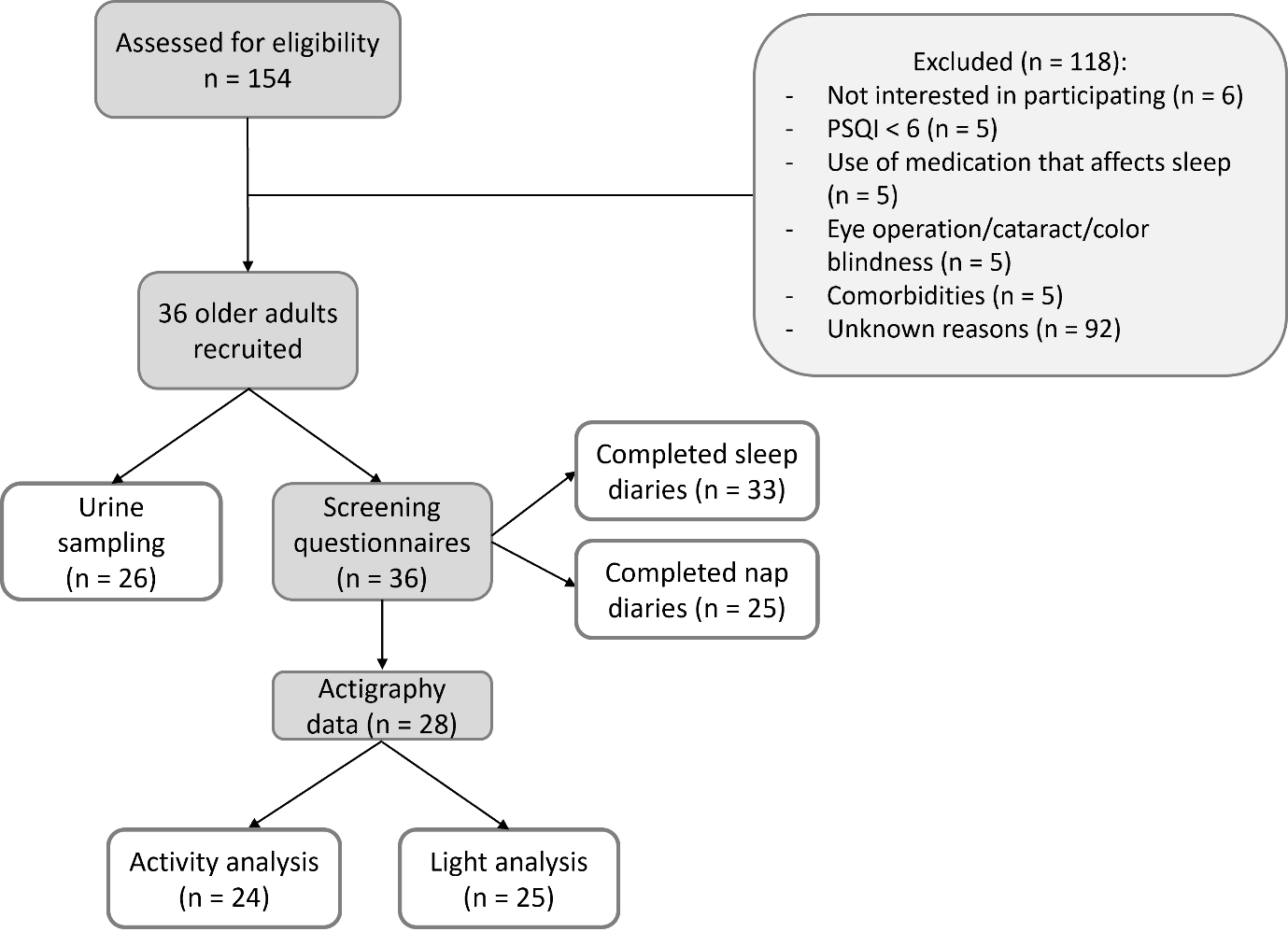


**Fig.S1. CONSORT diagram.** Grey squares indicate the intermediate number, and white squares indicate the final sample sizes for this study.

**Light Characteristics**

The light sources used consisted of flat light boxes containing 17000 K fluorescent bulbs (*Master PL-L 36W/840/4P)* for the blue-enriched white light condition and 4000 K fluorescent bulbs *(Master PL-L 36/865/4P)* for the control white light condition (*Fig.2a*). Light conditions were designed to mimic a skeleton photoperiod to provide a stronger lights-on lights-off signal to the clock simulating a complete photoperiod of a 12:12 light-dark cycle. These were provided by Philips Lighting, Eindhoven, The Netherlands. The two light conditions were set at a similar irradiance and photon density (*Fig.2b*). Since ocular photopigments are sensitive to the number rather than the energy of photons emitted by a light source, maintaining a similar total number of photons is essential when comparing the biological effectiveness of different lights. Thus, the major difference between the light sources was the amount of photons contained within the short-wavelength blue region (*Fig.2c*) in which the blue-enriched light emitted approximately 1.5 fold more short-wavelength photons (400-490 nm) than the control light. Fifteen participants were exposed to low light intensity (300-450 lux), while 21 participants were exposed to high light intensity (1100-1200 lux). Comparison between the low and high intensity groups made it possible to measure whether differences in the magnitude of responses could be attributed to the differences in spectral composition or irradiance. The lamps used in this study met ultraviolet (UV) and blue light hazard safety standards (standard ICNIRP 7/99 and CIE S 009/E: 2002).

| **Circadian phase assessment – Radioimmunoassay (RIA):**  To assess circadian phase, participants collected 11 sequential urine samples over 39 h comprising two nights and the day in between (starting from 21:00 h on day 1 until 12:00 h on day 3). The two overnight collections were from bedtime until wake-up/get-up time (ca. 8-h each) whereas the daytime collection was split into 3-hour intervals. For each of the 11 urine samples collected by each participant, a 5 ml aliquot was kept at 4°C or below in the participants’ home fridge. A researcher then collected the samples and these were stored at -20°C at the University of Surrey until analysis. The major urinary metabolite of melatonin, 6-sulphatoxymelatonin (aMT6s), was measured to provide an indirect assessment of SCN circadian phase. aMT6s was measured using a radioimmunoassay (RIA) procedure [1]. From that the aMT6s concentrations were calculated using the “RIAcalc” software program and the aMT6s standard curve. The aMT6s acrophase (peak time) was calculated using cosinor analysis, which is a curve-fitting procedure that assumes the time series is sinusoidal [2].  **TABLE S1.** Participants’ self-reported light condition preference. | | | |
| --- | --- | --- | --- |
| **LOW IRRADIANCE (n = 15)** | | **HIGH IRRADIANCE (n = 21)** | |
| **Blue-enriched light** | **Control light** | **Blue-enriched light** | **Control light** |
| “makes eyes uncomfortable” | “left eye hurt a bit in the beginning” | “eyes sore after each session, did not like this light but sleep better with this light” | “possibly better sleep with 1^st^ (control) light” |
| “(blue) far better” | “made improvement” | “stopped blue light because of migraines” | “more effective than 1^st^ (blue) light (energy)” |
| “this is better light, nicer to look at” | “a bit burning of the eyes when tired, but sleep may be better” | “slight headaches with the light” | “helped with migraines” |
| “makes me feel awake” | “a bit intrusive” | “better, friendlier, calming effect” | “this may be better” |
| “(blue) better despite glare at beginning of it” |  | “(blue) better despite glare at beginning of it” |  |

**
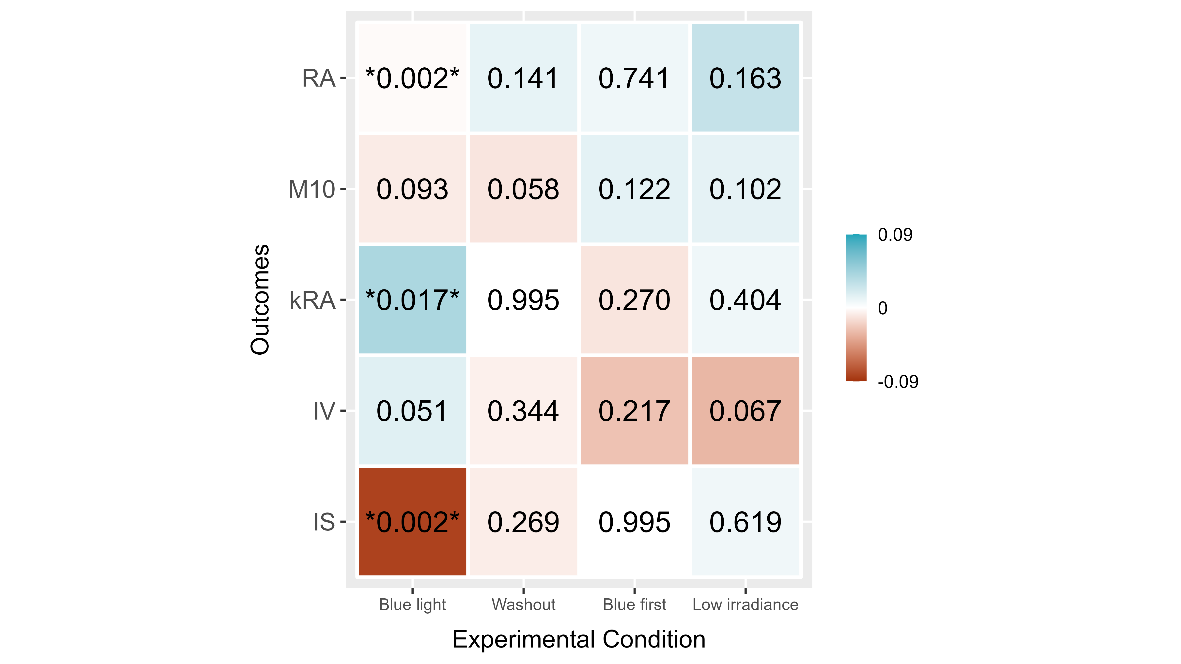
**

**Fig.S2. Heatmap illustrating the associations between outcomes and fixed variables derived from ANOVA models.** The intensity of colors within each square corresponds to the estimated effect size (darker shades represent larger effect sizes, whereas lighter shades represent smaller effect sizes). The numerical values inside the squares indicate the associated p-values, with p-values < 0.05 shown between “*”. RA, relative amplitude; kRA, sleep fragmentation; IV, intradaily variability; IS, interdaily stability.

| **Table S2.** Repeated measures ANOVA results with relative amplitude (RA) as the outcome. | | | | | | | |
| --- | --- | --- | --- | --- | --- | --- | --- |
|  | **Estimate** | **Std. Error** | **df** | **t-value** | **F** | **Sig.** | **95% Confidence interval** |
| **(Intercept)** | -0.025 | 0.07 | 18.514 | -0.344 | 0.096 | 0.735 | [-0.18, 0.13] |
| **Light condition = Blue** | -0.004 | 0.01 | 20.596 | -0.637 | 9.868 | 0.531 | [-0.02, 0.01] |
| **Washout = 0** | 0.001 | 0.01 | 38.383 | 0.092 | 0.008 | 0.927 | [-0.01, 0.06] |
| **Blue first** | -0.007 | 0.02 | 19.380 | -0.320 | 0.103 | 0.752 | [-0.05, 0.04] |
| **Intensity level (Low)** | 0.028 | 0.02 | 19.374 | 1.580 | 1.029 | 0.130 | [-0.01, 0.06] |
| **Blue*Low intensity** | -0.021 | 0.01 | 18.000 | -2.537 | 6.436 | **0.021** | [-0.04, 0.00] |
| **TAT 250 lux** | 0.000 | 0.00 | 40.561 | 1.857 | 3.450 | 0.071 | [-0.00, 0.00] |
| **Baseline RA** | 0.992 | 0.09 | 18.398 | 10.456 | 109.322 | **<.001** | [0.79, 1.19] |
| TAT 250 lux = Time spent above 250 lux (min) | | | | | | | |

| **Table S3.** Repeated measures ANOVA results with M10 as the outcome. | | | | | | | |
| --- | --- | --- | --- | --- | --- | --- | --- |
|  | **Estimate** | **Std. Error** | **df** | **t-value** | **F** | **Sig.** | **95% Confidence interval** |
| **(Intercept)** | 0.17 | 0.09 | 14.12 | 2.00 | 4.334 | 0.065 | [-0.01, 0.36] |
| **Light condition = Blue** | 0.00 | 0.00 | 17.41 | -0.93 | 0.864 | 0.365 | [-0.02, 0.01] |
| **Washout = 0** | -0.02 | 0.01 | 35.62 | -2.23 | 4.994 | **0.032** | [-0.03, 0.00] |
| **Blue first** | 0.01 | 0.01 | 15.22 | 0.97 | 0.937 | 0.348 | [-0.01, 0.03] |
| **Intensity level (Low)** | 0.02 | 0.01 | 16.88 | 2.33 | 5.429 | **0.032** | [0.00, 0.04] |
| **Number naps** | -0.01 | 0.00 | 40.80 | -4.51 | 20.321 | **<.001** | [-0.01, 0.00] |
| **TAT 250 lux** | 0.00 | 0.00 | 44.76 | 1.79 | 3.197 | 0.081 | [0.00, 0.00] |
| **Baseline M10** | 0.81 | 0.09 | 14.24 | 8.62 | 74.318 | **<.001** | [0.61, 1.01] |
| TAT 250 lux = Time spent above 250 lux (min). | | | | | | | |

**
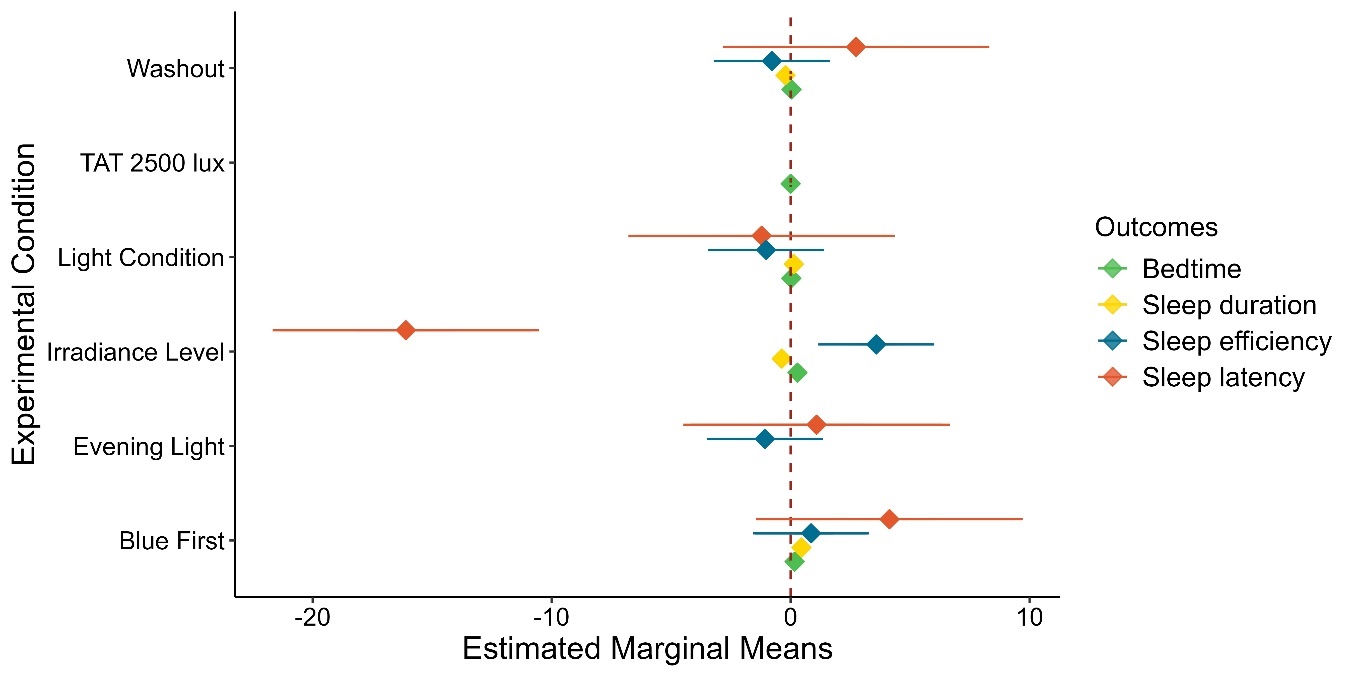
**

**Fig.S3.** Forest plot illustrating the estimated effect sizes of individual independent variables associated with the subjective sleep measures.

**Table S4.** Repeated measures ANOVA results for subjective measures of sleep.

| **BEDTIME** | **Estimate** | **Std. Error** | **df** | **t-value** | **F** | **Sig.** | **95% Confidence interval** |
| --- | --- | --- | --- | --- | --- | --- | --- |
| **(Intercept)** | 0.099 | 2.29 | 23.03 | 5.07 | 26.99 | **<.001** | [6.87, 16.33] |
| **Light condition = Blue** | 0.027 | 0.05 | 24.32 | 1.76 | 3.09 | 0.091 | [-0.01, 0.15] |
| **Washout = 0** | 0.038 | 0.03 | 28.34 | 1.33 | 1.78 | 0.193 | [-0.02, 0.11] |
| **Blue first** | 0.168 | 0.19 | 23.07 | 1.07 | 1.16 | 0.294 | [-0.19, 0.60] |
| **Intensity level (Low)** | 0.288 | 0.20 | 23.04 | 1.46 | 2.12 | 0.159 | [-0.12, 0.69] |
| **TAT 2500 lux** | 0.000 | 0.00 | 52.44 | -2.61 | 6.81 | **0.012** | [-0.00, 0.00] |
| **Baseline bedtime** | 0.488 | 0.10 | 23.02 | 4.91 | 24.14 | **<.001** | [0.28, 0.69] |
| **SLEEP LATENCY** | **Estimate** | **Std. Error** | **df** | **t-value** | **F** | **Sig.** | **95% Confidence interval** |
| **(Intercept)** | -38.140 | 21.74 | 25.94 | -1.75 | 4.07 | 0.090 | [-82.84, 6.56] |
| **Light condition = Blue** | -1.212 | 1.10 | 27.96 | -0.97 | 0.58 | 0.342 | [-2.65, 0.95] |
| **Washout = 0** | 2.742 | 2.04 | 28.29 | 1.36 | 1.80 | 0.185 | [-1.41, 6.97] |
| **Blue first** | 4.138 | 5.25 | 24.94 | 0.79 | 0.62 | 0.434 | [-6.63, 14.96] |
| **Intensity level (Low)** | -16.099 | 5.58 | 25.07 | -2.82 | 8.09 | **0.009** | [-26.73, -4.15] |
| **Evening light exposure** | 1.087 | 0.54 | 25.34 | 2.02 | 4.09 | 0.054 | [-0.02, 2.19] |
| **Baseline sleep latency** | 0.742 | 0.07 | 25.22 | 10.90 | 118.99 | **<.001** | [0.60, 0.88] |
| **SLEEP DURATION** | **Estimate** | **Std. Error** | **df** | **t-value** | **F** | **Sig.** | **95% Confidence interval** |
| **(Intercept)** | 5.363 | 0.90 | 27.07 | 5.95 | 36.80 | **<.001** | [3.51, 7.21] |
| **Light condition = Blue** | 0.135 | 0.08 | 29.16 | 0.97 | 0.93 | 0.342 | [-0.07, 0.19] |
| **Washout = 0** | -0.214 | 0.05 | 30.15 | -4.41 | 19.44 | **<.001** | [-0.31, -0.11] |
| **Blue first** | 0.453 | 0.29 | 27.27 | 1.55 | 2.39 | 0.134 | [-0.14, 1.03] |
| **Intensity level (Low)** | -0.367 | 0.28 | 27.13 | -1.59 | 2.51 | 0.124 | [-0.97, 0.12] |
| **Baseline sleep duration** | 0.306 | 0.11 | 27.00 | 2.71 | 7.33 | **0.012** | [0.07, 0.54] |
| **SLEEP EFFICIENCY** | **Estimate** | **Std. Error** | **df** | **t-value** | **F** | **Sig.** | **95% Confidence interval** |
| **(Intercept)** | 76.666 | 12.18 | 18.84 | 6.29 | 45.43 | **<.001** | [51.17, 102.16] |
| **Light condition = Blue** | -1.019 | 0.92 | 22.38 | -0.18 | 0.03 | 0.860 | [-1.82, 1.53] |
| **Washout = 0** | -0.787 | 0.67 | 24.58 | -0.74 | 0.55 | 0.467 | [-1.86, 0.88] |
| **Blue first** | 0.856 | 2.36 | 19.14 | 0.31 | 0.10 | 0.758 | [-3.66, 4.95] |
| **Intensity level (Low)** | 3.585 | 2.43 | 19.34 | 3.13 | 9.82 | **0.005** | [2.11, 10.55] |
| **TAT 2500 lux** | 0.002 | 0.00 | 44.12 | 2.59 | 6.72 | **0.013** | [0.00, 0.00] |
| **Evening light exposure** | -1.075 | 0.23 | 19.27 | -4.76 | 22.62 | **<.001** | [-1.56, -0.61] |
| **Baseline sleep efficiency** | 0.597 | 0.09 | 20.26 | 6.39 | 40.82 | **<.001** | [0.40, 0.79] |


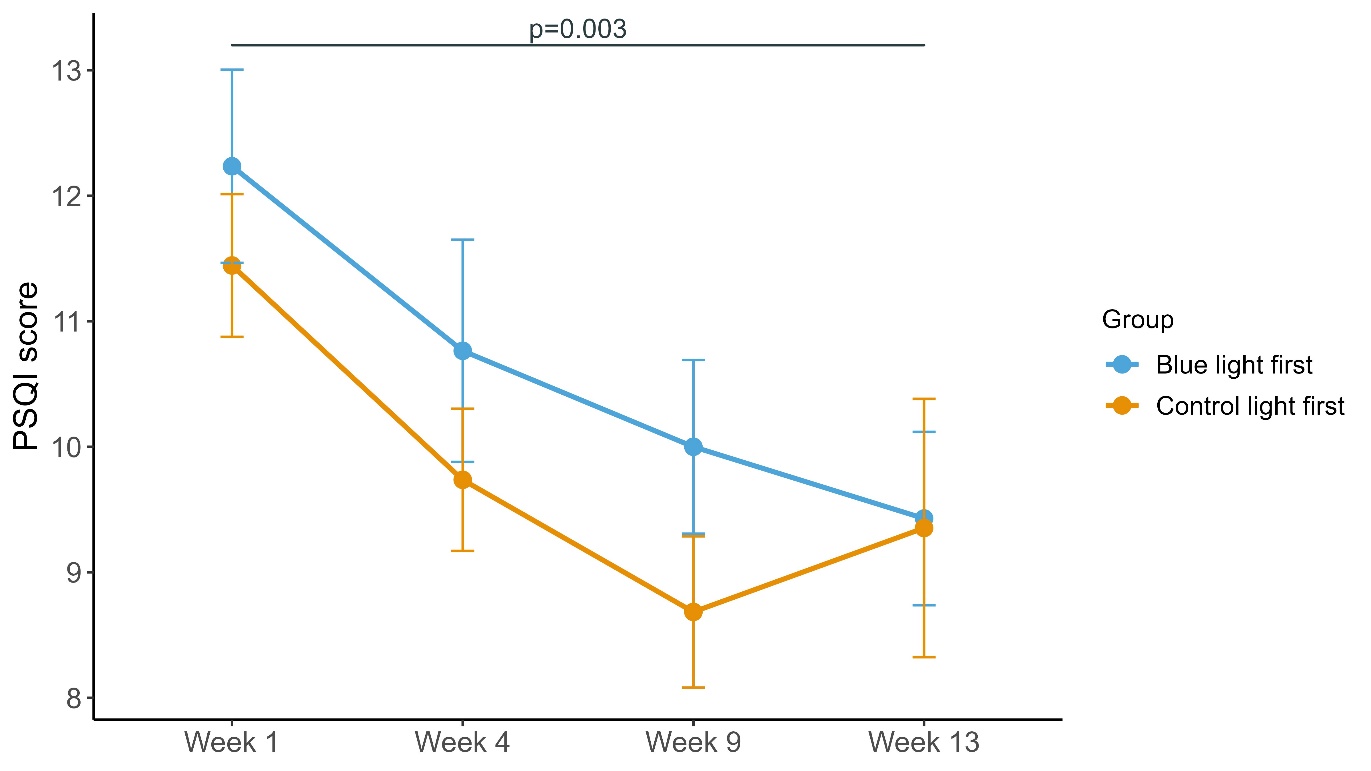


**Fig.S4.** Mean PSQI score (± SE) over the study weeks for both groups: group that received blue-enriched light first (blue line) and the group that received control light first (orange line). A paired t-test indicated a significant difference (p-value = 0.003) between baseline (week 1) PSQI score and PSQI on week 13 (2 weeks post-protocol completion) Higher PSQI score indicates poorer sleep quality.

**
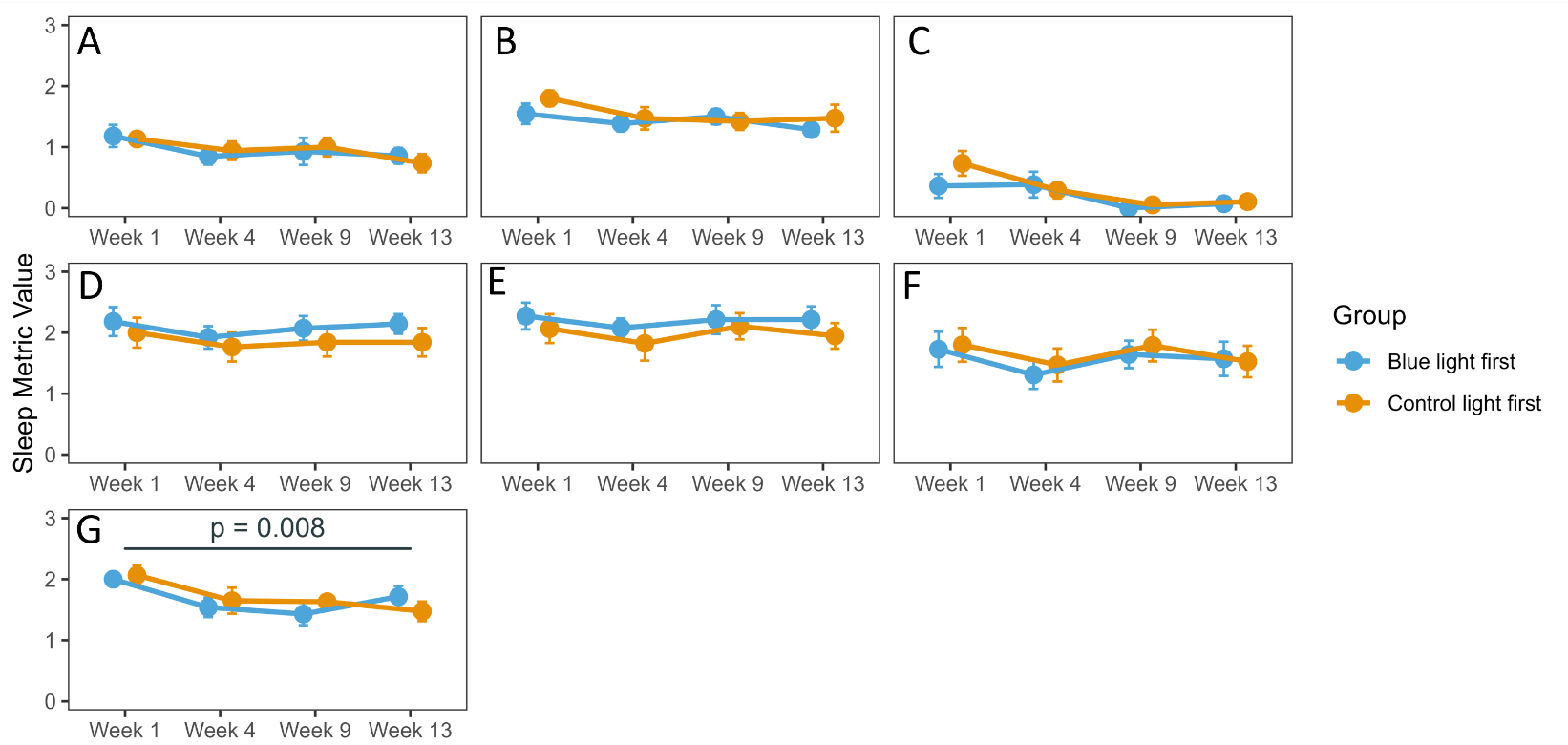
Fig.S5.** Mean score (± SE) for each PSQI component over the study weeks for the group that received blue-enriched light first (blue line) and the group that received control light first (orange line). **a:** Daytime dysfunction; **b:** Sleep disturbances; **c:** Sleep drugs; **d:** Sleep duration; **e:** Sleep efficiency; **f:** Sleep latency; **g:** Sleep quality. A paired t-test indicated a significant difference only in the sleep quality component which was significantly improved (p = 0.008) from baseline (Week 1) to after the study end (Week 13).

**REFERENCES**

[1] Aldhous ME, Arendt J. Radioimmunoassay for 6-sulphatoxymelatonin in urine using an iodinated tracer. Ann Clin Biochem. 1988;25(3):298-303. <https://doi.org/10.1177/000456328802500319>

[2] Nelson W, Tong YL, Lee JK, Halberg F. Methods for cosinor-rhythmometry. Chronobiologia. 1979;6(4):305-323.
